# Genomic Insights into *Aquimarina* sp. Strain EL33, a Bacterial Symbiont of the Gorgonian Coral *Eunicella labiata*

**DOI:** 10.1128/genomeA.00855-16

**Published:** 2016-08-18

**Authors:** Tina Keller-Costa, Rúben Silva, Asunción Lago-Lestón, Rodrigo Costa

**Affiliations:** a Microbial Ecology and Evolution Research Group, Centre of Marine Sciences (CCMar), University of Algarve (UAlg), Gambelas, Faro, Portugal; b Departamento de Innovación Biomédica, Centro de Investigación y Estudios Superiores de Ensenada (CICESE), Ensenada, Baja California, Mexico; c Department of Bioengineering, Institute for Bioengineering and Biosciences (IBB), Instituto Superior Técnico, Universidade de Lisboa, Lisbon, Portugal

## Abstract

To address the metabolic potential of symbiotic *Aquimarina* spp., we report here the genome sequence of *Aquimarina* sp. strain EL33, a bacterium isolated from the gorgonian coral *Eunicella labiata*. This first-described (to our knowledge) animal-associated *Aquimarina* genome possesses a sophisticated repertoire of genes involved in drug/antibiotic resistance and biosynthesis.

## GENOME ANNOUNCEMENT

The recently described *Aquimarina* genus (1), so far comprising 20 recognized species, encompasses primarily marine heterotrophic aerobic bacteria belonging to the metabolically plastic *Flavobacteriaceae* family (*Bacteroidetes*). Currently, there are only 10 *Aquimarina* genome sequences available in public databases (http://www.ncbi.nlm.nih.gov), all describing planktonic or alga-associated strains. *Aquimarina* species have also been retrieved from several animal hosts (2–5), but knowledge of their roles in association with metazoans is limited. To enable an inspection of the putative symbiotic features of the genus, we announce the nearly complete genome sequence of *Aquimarina* sp. strain EL33, a strain isolated from the gorgonian coral *Eunicella labiata* Thomson 1927. The host organism was sampled at ca. 18-m depth in the Atlantic Ocean, offshore of the Algarve region, South Portugal (36°58′47.2ʺN, 7°59′20.8ʺW). In the laboratory, host-derived microbial cell suspensions were prepared as described previously (6) and inoculated onto diluted (1:2) marine agar medium for 1 week at 18°C. Genomic DNA of strain EL33 was extracted and sequenced on an Illumina MiSeq platform, as performed elsewhere (7, 8). Sequence output was about 1.02 Gb, comprising 2 × 1,701,863 paired-end reads of 301 bp, corresponding to ca. 163× coverage of the genome. Sequence reads were assembled *de novo* into 20 contigs with the NGen DNA assembly software by DNAStar, Inc. Gene prediction and annotation were performed with the Rapid Annotation using Subsystem Technology (RAST) prokaryotic genome annotation server, version 2.0 (9).

The genome is composed of 6,270,711 bp, with a calculated G+C content of 32.9%. It possesses 5,530 coding sequences in addition to 40 tRNA and five rRNAs. *Aquimarina* sp. EL33 presents the highest 16S rRNA gene similarity (99.7%) with *Aquimarina* sp. Aq349, isolated from the marine sponge *Sarcotragus spinosulus* (6). *Aquimarina megaterium* XH134, isolated from surface seawater (10), is the closest described type strain (99.2% 16S rRNA gene similarity).

*Aquimarina* sp. EL33 possesses several genes involved in nutrient cycling (C, N, and S), suggesting versatile nutrient acquisition and utilization, in line with observations made for *Aquimarina longa* SW024 (11). For example, 19 chitinase-encoding genes (endochitinases EC 3.2.1.14) were found, indicating that strain EL33 is capable of degrading chitin, the most abundant polysaccharide in the oceans. Indeed, *in vitro* chitinolytic activity was shown for *A. longa* SW024 (11), which possesses seven chitinase-encoding genes.

The genome further reveals an elaborate arsenal of defense mechanisms. Thirty-five β-lactamase-encoding genes were identified, suggesting resistance of the strain to manifold β-lactam antibiotics. In addition, several genes encoding multidrug efflux pumps, drug transporters, and transition-metal cation binding proteins were detected, possibly enabling strain EL33 to cope with the activity of competing microorganisms and to persist in/on its host. Highlighting the potential antimicrobial activity of *Aquimarina* sp. EL33 is the presence of the *lodAB* operon responsible for the biosynthesis of marinocine, a lysin oxidase antimicrobial protein. Also, we observed at least two polyketide synthase (PKS)-encoding genes in the EL33 genome, corroborating earlier PCR-based detection of PKS genes across several strains of the genus (6).

### Accession number(s).

The genome sequence of *Aquimarina* sp. EL33 has been deposited in the European Nucleotide Archive (ENA) under the accession numbers FLRG01000001 to FLRG01000020. The study Identification number is PRJEB14417.
